# Assessment of Perinatal Care Satisfaction Amongst Mothers Attending Postnatal Care in Ibadan, Nigeria

**DOI:** 10.29024/aogh.10

**Published:** 2018-04-30

**Authors:** Titilayo Dorothy Odetola, Emmanuel Oluwabukunmi Fakorede

**Affiliations:** 1 Department of Nursing, University of Ibadan, Nigeria, NG

## Abstract

**Introduction:**

Patient satisfaction has been identified as a major index in the assessment of quality of healthcare globally. Mothers judge the quality of perinatal care received based on their satisfaction with the services provided, thus influencing their utilization of the available health facilities. There is currently a dearth of literature on users’ satisfaction of services rendered at the primary level of care, which is the first port of call to the health system aimed at serving majority of the populace.

**Objectives:**

The study was set out to investigate maternal satisfaction with perinatal care received in selected primary health centres in Ibadan.

**Methods:**

The study was a cross-sectional survey involving 66 women receiving postpartum care from five randomly selected primary health centres in Ibadan north-west local government using a 72-itemed questionnaire with p ≤ 0.05.

**Findings:**

The majority of the respondents (98.5%) perceived the perinatal care they received as high quality, and 94% and 98% were satisfied with services and facilities used for their care, respectively. Identified causes of dissatisfaction included dirty hospital environment, inadequate water supply and hospital facilities, distance of hospital location, cost of materials, time wasting, inadequate staffing and poor attitude, and verbal and physical abuse. An association exists between maternal satisfaction with quality of care and future intention for subsequent utilization (χ^2^ = 13.306; p = 0.0001).

**Conclusion:**

The overall maternal satisfaction on the perinatal care provided was perceived as very good. However, few domains of dissatisfaction were identified that would need to be addressed by healthcare professionals and policymakers to sustain and improve utilization of orthodox health care services amongst mothers, thereby contributing to achieving the third Sustainable Development Goal.

## Introduction

Patient satisfaction can be defined as patients’ judgement on the quality and goodness of care [1]. It means the best health outcomes that are possible given the available resources and should be consistent with patients’ values and preferences. It is essential to point out that satisfaction of patients is an integral element of health status and constitutes a measure of the outcome of care used in evaluating distinct dimensions of patients’ healthcare [2]. Although there is no universally accepted method of measuring quality of care, there is growing consensus that measuring quality of care should be based at least on patients’ satisfaction studies [3,4].

The quality of perinatal healthcare in most developing countries is chronically poor and grossly appalling [5,6]. This has been identified as one of the precursors to the unacceptably high maternal mortality rate in developing countries [7].

The World Health Organization reported that approximately 830 women die from preventable causes related to pregnancy and childbirth every day, with 99% of all these maternal deaths occurring in developing countries [7]. In 2013, Nigeria was ranked third amongst countries with highest maternal mortality, with Nigeria and India alone stated to be responsible for one-third of total maternal deaths worldwide [8]. According to the Maternal Mortality Estimation Inter-Agency group (MMEIG), maternal mortality rate in Nigeria was 560 per 100,000 live births in 2013 [9]. A woman’s lifetime risk of maternal death – the probability that a 15-year old woman will eventually die from a maternal cause – is 1 in 4,900 in developed countries versus 1 in 180 in developing countries [7]. In Nigeria, lifetime risk of death of pregnant women is 1 in 23 [10]. However, the high mortality rate reported in Nigeria has been linked to lack of quality perinatal care within the country [5]. It has been further emphasized that there is a clear link between poor quality of perinatal care, maternal dissatisfaction with care, poor usage of health care facilities, and increased maternal mortality rate in regions where such precursors exists [7].

The primary target of the United Nations’ Sustainable Development Goal 3 is to reduce the global maternal mortality rate to less than 70 per 100,000 live births, with no countries having a maternal mortality rate higher than twice the global average [11]. A major effort being made to achieve this is to ensure equal and universal accessibility to quality health care services, which can only be achieved through primary health care. Hence, it is essential to pay more attention to the quality of perinatal services provided at these PHCs if this primary target of SDG3 is to be accomplished [11]. Because primary health care ensures equal and universal accessibility to quality health care services, it is important to note that the provision of adequate primary health care is essential to improving the general health status globally.

However, in most developing countries, the quality of primary health care services available is very poor [5]. The primary health care centres available are plagued with so many problems such as inadequate funding, lack of trained personnel, lack of essential resources, poor attitudes of healthcare workers, and lack of community participation, amongst others [4]. One of the major services provided at these primary health centres is perinatal care. However, it has been reported that some women are dissatisfied with the quality of services obtained at these centres, with report of poor attitudes of healthcare workers being a major cause of dissatisfaction [1,12].

Poor attitude of caregivers such as ineffective communication (impolite, frowning, whispering, not explaining procedures), neglect (not attending to clients promptly, not involving client in the care), unfriendliness (shouting, ignoring), and financial factors (no explanation on hospital bills), amongst others, are major factors that makes women uncomfortable [12]. This results in lack of maternal satisfaction with perinatal care and subsequent non-utilization of the hospital in the future, or using the hospital only as a last resort resulting in increased maternal mortality [12,1].

The majority of people who opt for perinatal care at these primary health centres are often women within low socio-economic class who accept whatever services offered. Thus poor quality healthcare services received is often underreported and only expressed by non-usage of such a facility in subsequent times – defeating the purpose of primary health care, which is to provide quality health care for all [13,14].

The health care system in Nigeria has received numerous negative comments both from the patients and the society [15]. These negative comments range from poor quality of service delivery to service delay, discontinuity of care, indifferent staff attitude, and bureaucratic procedures [12]. These negative comments have led to poor public confidence in healthcare and made the government hospitals unattractive to the consumers of hospital services [15,12]. Quality of care requires that healthcare providers constantly check whether the care offered is effective, humane, and patient centred, and that the health consumer’s expectation and needs are satisfied. This is a professional responsibility owed generally to the consumers of healthcare goods and services.

There may be evidence of the success in providing more primary health centres recently against what used to be in Nigeria before, but if they are not being tailored to clients’ needs and satisfaction, they are of no use to the clients. The main focus is to meet the clients’ (perinatal women) needs and expectations as a means of attaining objectives of improving maternal and child health and incorporating periodic patient satisfaction studies into the quality improvement plan in the service delivery in selected primary health centres.

Most studies on satisfaction with quality of healthcare services has been in the higher levels of the health facilities and with broader focus, but this study is focused on assessing maternal satisfaction with perinatal care at the primary healthcare level, which is actually the first port of call to the health system and which is expected to serve majority of the populace. Satisfaction with perinatal care services may well be a pointer to general service of the entire primary health care (PHC) facility.

It is against this background that the researcher set out to assess perinatal women’s satisfaction with quality of care received at selected primary health centres using selected indices of satisfaction in the primary health centres. Specific objectives included (a) to assess perception of study participants regarding quality of perinatal care received at PHC; (b) to assess level of satisfaction of study participants with perinatal nursing care received at selected primary health centres; (c) to examine the relationship between patients’ expectation and satisfaction with perinatal nursing care; (d) to examine the relationship between maternal satisfaction with perinatal care and subsequent intention of utilization of primary health centres for perinatal care; and (e) to examine factors associated with satisfaction or dissatisfaction of study participants.

## Methods

This study was a descriptive cross-sectional survey. It was designed to collect information on maternal satisfaction with perinatal care and its relationship with choice of place of birth for subsequent pregnancies. The survey was carried out in the 5 major primary healthcare centres in the Ibadan North-West Local Government Area, Oyo State, Nigeria. These facilities were chosen due to their location and proximity to the semi-literate and low socio-economic community in Ibadan, an urban community in south-west Nigeria. This method was considered appropriate because it gave quantitative information about the situation under study.

The study included all consenting women who had received antenatal and intranatal care, and were currently receiving postpartum care, at Oniyanrin PHC, Ayeye PHC, Orieru PHC, Eleyele PHC, and Koseunti community PHC. Women who were receiving postpartum care but who did not receive antenatal and intranatal care at the same primary health centre were excluded from this study. Also, women who did not consent to participating in the study and other women who were receiving care for other medical conditions at these primary health centres were excluded.

Using the Fisher’s sample size calculation formula, with consideration for attrition, a study population comprising of 66 women who consented and were receiving perinatal care and who had received antenatal and intranatal care at all the five major primary health centres in Ibadan North-West Local Government; Oniyanrin PHC, Ayeye PHC, Orieru PHC, Eleyele PHC, and Koseunti community PHC in Ibadan. New antenatal clients are registered every day of the week but with specific days for postnatal visits. Simple random sampling was used for this study.

A 72-itemed researcher-developed and self-administered structured questionnaire, which was well assessed for face and content validity, was used for the study. The instrument was also ascertained for stability, consistency, and equivalence by the use of the Kuder-Richardson (KR-20) formula to determine the internal consistency reliability coefficient of the instrument. A KR-20 score of 0.7 was obtained, which indicates a high level of internal consistency reliability.

An ethical approval was obtained from the UI/UCH Health Ethical Review Committee. Data was collected and processed using the Statistical Package for Social Sciences (SPSS) version 20.0. Descriptive statistics of frequency distribution tables, pie charts, and bar charts were used to describe the data.

## Findings

Table 1 shows the socio-demographic characteristics of the respondents. Findings revealed that the population of women who mainly received perinatal care in most PHCs in Ibadan North-West Local Government were married and between 20 and 29 years old. They had secondary school education, were self-employed, and were from the south-west region. This may be due to the fact that the research was conducted in the south-western part of the country. The majority of these women could read neither the English nor the Yoruba version of the questionnaire successfully. It may be safe to conclude that majority of women receiving perinatal care in PHCs in Ibadan North-West Local Government had a low level of education and mostly belong to the low socio-economic class in society.

**Table 1:** Socio-demographic Data.

Variable	Frequency	Percentage (%)

**1. Age (years)**		
16–19	8	12.1
20–24	20	30.3
25–29	22	33.3
30–34	10	15.2
35–39	4	6.1
40–44	2	3.0
**2. State of Origin**		
South-west	62	93.9
South-south	1	1.5
South-east	3	4.5
North-west	0	0
North-east	0	0
North-central	0	0
**3. Marital Status**		
Single	5	7.6
Married	61	92.4
Separated	0	0
Divorced	0	0
Widowed	0	0
**4. Level of Education**		
Primary School	7	10.6
Secondary School	44	66.7
Tertiary Education	15	22.7
**5. Occupation**		
Civil Servant	3	4.5
Private Institution Employee	9	13.6
Self-employed	46	69.7
Trader	4	6.1
Unemployed	4	6.1

Table 2 below shows a pattern of responses obtained to questions structured to elicit respondents’ perception of quality of perinatal care received. Sixty-four (64, 97%) respondents agreed that the quality of perinatal care they received was excellent and that they were happy with the way the midwives took care of them. Also, 64 respondents (97%) agreed that they believed the midwives were very competent, and 63 respondents (95.5%) submitted that the midwives and nurses had good attitude towards the patients. Over ninety percent (90.9%) agreed that the nurses were very respectful, and 62 respondents agreed that midwives always showed respect to their family members. Sixty-four (97%) respondents agreed that the midwives always explained their procedures; 62 respondents (93.9%) agreed that nurses always sought their consent before performing their procedures. Sixty respondents (90.9%) agreed that the midwives provided encouragement and support for them during labour and delivery, and 14 respondents submitted that they had once been beaten during delivery process by a nurse in the health centre.

**Table 2:** Respondents’ perception of quality of perinatal care received at PHC.

Variables	Yes	No

Overall, the quality of care provided here is excellent	**64(97%)**	**2(3%)**
I am happy with the way the midwives here take care of me	**64(97%)**	**2(3%)**
The midwives have been very nice to me	**63(95.5%)**	**3(4.5%)**
I received enough support from the nurses during antenatal care	**63(95.5%)**	**3(4.5%)**
I received enough support from the midwives during delivery	**62(93.9%)**	**4(6.1%)**
The midwives encouraged and supported me during labour	**60(90.9%)**	**6(9.1%)**
The midwives here always anticipate and pay attention to my healthcare needs	**64(97%)**	**2(3%)**
I believe the midwives here are very competent	**64(97%)**	**2(3%)**
The midwives here have good attitude towards the patients	**63(95.5%)**	**3(4.5%)**
The midwives provided encouragement and support for me during labour	**62(93.9%)**	**4(6.1%)**
The midwives here are very respectful	**60(90.9%)**	**6(9.1%)**
The midwives here speak to me very kindly	**62(93.9%)**	**4(6.1%)**
The midwives always explain their procedures to me before they perform them	**64(97%)**	**2(3%)**
The midwives here show respect to me and my family	**62(93.9%)**	**4(6.1%)**
The midwives here always seek my permission before they touch me	**62(93.9%)**	**4(6.1%)**
I was once beaten by a nurse in this hospital	**14(21.2%)**	**52(78.8%)**
I was once verbally abused by a nurse in this hospital	**16(24.2%)**	**50(75.8%)**

Table 3 below reports the level of maternal satisfaction with perinatal nursing care received in the primary health centres. A total of 60 respondents (90.9%) accepted that all their expectations about nursing care were met, 80.3% stated that they actually received better care than they had expected, and 89.4% stated that the nurses and midwives performed to their expectation. Ninety-seven percent (97%) consented that they were satisfied with the quality of antenatal care they had received, 92.4% agreed that they were satisfied with the care they received during labour, and 61 respondents stated that the postpartum care they were currently receiving is satisfactory.

**Table 3:** Satisfaction with perinatal nursing care.

Satisfaction with perinatal nursing care	Yes(%)	No(%)

Overall, all my expectations about nursing care were met	**60(90.9%)**	**6(9.1%)**
I received better care than I expected	**53(80.3%)**	**13(19.7%)**
The nurses and midwives performed to my expectation	**59(89.4%)**	**7(10.6%)**
I am satisfied with the postpartum care I am currently receiving at this hospital	**61(92.4%)**	**5(7.6%)**
I am happy with the quality of antenatal care I received in this hospital	**64(97%)**	**2(3%)**
I was satisfied and comfortable with giving birth in this hospital	**63(95.5%)**	**3(4.5%)**
I received adequate pain management when I had my baby in this hospital	**57(86.4%)**	**9(13.6%)**
I was satisfied with the care rendered to me during labour in this hospital	**61(92.4%)**	**5(7.6%)**
The midwives are skilful and competent	**63(95.5%)**	**3(4.5%)**

Table 4 below reports women’s satisfaction with other aspects of care such as hospital environment, facilities, other hospital staffs, process of care, and affordability of care. Findings show that 64 respondents (97%) agreed that the hospital environment is neat and conducive, whereas 2 respondents (3%) stated otherwise. Sixty-two respondents agreed that the hospital has the facilities needed to provide perinatal care. In contrast, 45 respondents (68.2%) testified that nurses always complained of lack of instruments, although 21 respondents (31.8%) disagreed.

**Table 4:** Satisfaction with other aspects of care.

Satisfaction with other aspects of care	Yes(%)	No(%)

The hospital environment is neat and conducive	**64(97%)**	**2(3%)**
The hospital has all the facilities needed for my care	**62(93.9%)**	**4(6.1%)**
The nurses always complain of lack of instruments	**45(68.2%)**	**21(31.8%)**
I always have to buy things that the hospital is supposed to supply	**35(53%)**	**31(47%)**
There is adequate electricity and water supply to this hospital	**53(80.3%)**	**13(19.7%)**
There are good beds and bed sheets in the wards	**64(97%)**	**2(3%)**
There are good toilets and bathroom in this hospital	**61(92.4%)**	**5(7.6%)**
The hospital staffs attend to me as fast as possible	**61(92.4%)**	**5(7.6%)**
The hospital staffs address me with respect	**62(93.9%)**	**4(6.1%)**
I can afford to pay for the drugs and all services being provided in this hospital	**62(93.9%)**	**4(6.1%)**
I am satisfied with the facilities used for my care	**65(98.5%)**	**1(1.5%)**

Also, 35 respondents (53.3%) stated that they always had to buy things that the hospital was supposed to supply, but 31 respondents (47%) disagreed to paying out of pocket. Fifty-three respondents (80.3%) agreed to adequate electricity and water supply in the primary health centres, whereas about 90% of the respondents indicated satisfaction with the beds and bedsheets in the wards, the toilet and bathroom, attitude of staff, and cost of drugs and other medical services. Finally, 98% (65 respondents) indicated that they were satisfied with facilities used for their care.

Table 5 above indicates that based on the level of satisfaction with care received, 95.5% of the respondents would likely patronize the PHCs in Ibadan North-West for perinatal care for their subsequent pregnancies. Also, 98.5% (65 respondents) indicated that they would recommend the PHC to other women for perinatal care. Sixty-four respondents (97%) indicated that the care they had received is satisfactory.

**Table 5:** Effect of satisfaction with perinatal services with subsequent utilization of facility.

Effect of satisfaction with perinatal services with subsequent utilization of facility	Yes(%)	No(%)

Based on the quality of care you received here, would you recommend this facility to anyone you know?	**65(98.5%)**	**1(1.5%)**
Based on the quality of care you received here, would you visit this health centre to deliver your subsequent pregnancies?	**63(95.5%)**	**3(4.5%)**
Based on the quality of care you received here, would you say that the healthcare provided is satisfactory?	**64(97%)**	**2(3%)**

In summary, 75.8% of the respondents have high likelihood of subsequent patronage of the respective PHC for perinatal care, whereas 24.2% have low likelihood for subsequent patronage of PHC for perinatal care.

## Results of the Qualitative Aspect of Research Tool

The research tool (questionnaire) had both quantitative and qualitative aspects. Questions 67–72 on the research tool were structured to obtain qualitative information about satisfaction with care and likelihood for subsequent utilization of facility. Respondents were required to state the most important reason for their satisfaction or dissatisfaction, and they were required to identify the problems about the hospital as well as the things they appreciate most about the hospital.

**Figure 1:**
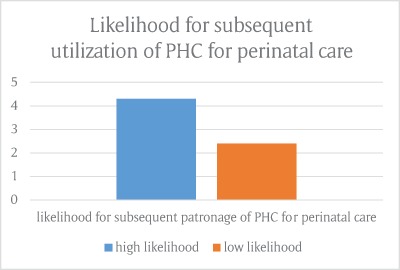
Likelihood for subsequent utilization of PHC for perinatal care.

On a scale of 10, respondents were required to rate the extent to which their expectations were met, and a mean score of 7 was obtained. A mean score of 8 was obtained when respondents were requested to rate the likelihood of subsequent delivery at the same PHC and a mean score of 9 was obtained when respondents were asked to rate the likelihood of recommending the PHC (Figure 1) to a friend.

Respondents perceived their nurses as hardworking and highly competent. The respondents stated, *“Nobody is perfect, but they [nurses] are okay.”* Other respondents stated, *“I gave them the score because they [nurses] tried enough for me; they did their best,”* and, *“I gave them the score because they [nurses] treat us kindly and always pray for us [women receiving perinatal care].”*

**Hypotheses Testing:** Relationship between perceived quality of care and women’s satisfaction with perinatal care.

As reported in Table 6, the results show that the variables were statistically significant. The p-value was less than 0.05, and hence the researchers rejected the hypothesis that stated that there is no relationship between perceived quality of care and women’s satisfaction with perinatal care.

**Table 6:** Perceived quality of care and women’s satisfaction with perinatal care.

Perceived Quality of Perinatal Care	Satisfaction with Perinatal Care		Total	χ^2^ value	p-value

	Good Satisfaction	Poor Satisfaction			

High Quality	62	3	65	15.738	0.00
Low Quality	0	1	1	
Total	62	4	66	

**Hypothesis 2:** Relationship between maternal level of education and women’s satisfaction with perinatal care.

Table 7 reports a statistically non-significant association between maternal education and satisfaction with care. The null hypothesis was accepted at p >0.05.

**Table 7:** Maternal level of education with maternal satisfaction with perinatal care.

Level of Education	Satisfaction with Perinatal Care		Total	χ^2^ value	p-value

	Good Satisfaction	Poor Satisfaction			

Primary	6	1	7	1.019	0.601
Secondary	42	2	44		
Tertiary	14	1	15		
Total	66	4	66		

**Hypothesis 3:** Relationship between maternal satisfaction with perinatal care and likelihood for subsequent patronage of primary health centres for perinatal care.

Table 8 reports the statistical association between perceived quality of perinatal care and likelihood for subsequent patronage. The results show that the variables were statistically significant. The p-value was less than 0.05, and hence we reject the hypothesis that states that there is no relationship between perceived quality of perinatal care and likelihood for subsequent patronage.

**Table 8:** Perceived quality of perinatal care with likelihood for subsequent patronage.

Perceived Quality of Perinatal Care	Likelihood for Subsequent Patronage		Total	χ^2^ value	p-value

	High Likelihood	Low Likelihood			

High Quality	50	12	62	13.306	0.0001
Low Quality	0	4	4		
Total	50	16	66		

## Discussion of Findings

Maternal satisfaction and perception of quality of care is an extremely important but often neglected aspect of perinatal care services in Nigeria because there are no measures put in place for frequent assessment of this aspect of quality of care [16]. It has been well established that mothers’ perception of the quality of care and their satisfaction is paramount to subsequent utilization, indirectly contributing to status of maternal mortality and morbidity in the country.

This study was conducted in the five major government-owned primary health care facilities in a local government area, which is mainly constituted by semi-urban and relatively rural communities in south-western Nigeria. The result obtained from the socio-demographic data revealed that the population of women who mainly received perinatal care in most PHCs in the local government were married and between 20 and 29 years old. This result is similar to a research conducted by Abodunrin, Adeomi & Adeoye carried out amongst mothers attending infant welfare clinics in Orolu Local Government Area (LGA), a semi-urban area of Osun state in south-western Nigeria, where most of the respondents (144, 72.0%) were between 20 and 29 years of age as well [17].

Furthermore, it was also discovered that majority of the women had a low level of education, and most of them could successfully read neither the English questionnaires nor the Yoruba questionnaires, which is their native language, despite the fact that majority of the women claimed to have secondary school education. This is in concord with a study by Nnebue, Ebenebe, Adinma, Iyoke, Obionu, and Ilika on clients’ knowledge, perception, and satisfaction with quality of maternal health care services at the primary health care level in Nnewi, Nigeria, where it was also discovered that majority of the women attending the PHC had comparatively low levels of education [18]. The same pattern was observed in a study conducted on Bitew, Ayichiluhm, and Yilmam in north-west Ethiopia, where the researchers also discovered that the majority (73.1%) of the respondents had less than high school level of education, out of which 15.4% could neither read nor write [19]. It could probably be concluded that the reason why most satisfaction studies in semi-urban or rural communities have high report of high level of satisfaction could be due to the low level of education.

### Satisfaction with Perinatal Nursing Care

Overall, very few respondents (6%) expressed dissatisfaction with the perinatal nursing care provided at the primary health centres. The majority of the respondents (94%, 62 respondents) were satisfied with services provided. The women expressed satisfaction with certain aspects of care, particularly the skills and competence with which the midwives and nurses provide care. This result is similar to previous studies on client satisfaction in Nigeria, which also reported that over 80% of clients were satisfied with services provided [20,21]. This result indicates that nurses are doing a great job at the grassroots, and their efforts in maintaining good patients’ satisfaction should be encouraged by provision of adequate facilities needed to provide standard quality care.

### Satisfaction with Other Aspects of Care

Majority of the respondents were generally satisfied with other aspects of care such as hospital environment, facilities, other hospital staff, process of care, and affordability of care. Findings shows that 64 respondents (97%) agreed that the hospital environment is neat and conducive, whereas 2 respondents (3%) stated otherwise. Sixty-two respondents agreed that the hospital has the facilities needed to provide care. In contrast, 45 respondents (68.2%) testified that nurses always complain of lack of instruments. This implies that nurses could have been improvising so much that most patients do not discover that the hospital actually does not have enough facilities needed to provide quality care, hence making their complain of lack of instruments seemingly incomprehensible and needless to clients. This is a very common occurrence because nurses are great ward managers and often go the extra mile to improvise for needed facilities, especially nurses practicing in rural primary health centres.

Also, 35 respondents (53.3%) expressed dissatisfaction about the fact that they always have to buy things that the hospital is supposed to supply. Apparently, the power (electricity) and water supply in most PHCs is satisfactory because a majority (53 respondents, 80.3%) agreed to adequate electricity and water supply in the primary health centres, with only 13 respondents (19.7%) indicating inadequate power and water supply as a problem in the PHCs. Satisfaction with power and water supply could probably be a result of low expectations in terms of power and water supply in the country generally. About 90% of the respondents indicated satisfaction with the beds and bedsheets in the wards, the toilet and bathroom, attitude of other hospital staff, and cost of drugs and other medical services. Finally, 98% (65 respondents) indicated that they are satisfied with facilities used for their care.

### Perception of Quality of Perinatal Nursing Care

Results from this study revealed that the majority of the women (98.5%) perceived the perinatal nursing care they received at the primary health centres as of high quality. This result is similar to the findings of Asekun-olarinmoye, Bamidele, Egbewale, & Asekun-Olarinmoye, Ojofeitimi [20].

### Maternal Expectation

It has been established in literature that the relationship between expectation and actual experience determines satisfaction [22,23]. The results from this study revealed that majority of the mothers had high expectations of quality of care. As presented in, 98.5% of respondents had high expectation about quality of perinatal nursing care while only 1.5% had low expectation. Results from this study points to some specific aspects of perinatal care that women expect. The study revealed that most women expect the nurses to give adequate health education about pregnancy care, give reassurance about their baby’s health during antenatal visit, be given the opportunity to discuss how they feel, be treated with respect, and be attended to as early as possible. They expect to be involved in decision making regarding their management, and they expect nice and caring nurses. They expect nurses to praise, support, and give encouragement during labour. Women expect to have the lowest pain possible during labour, believing that nurses should explain procedures and obtain their consent before they carry them out. They expect to be educated and supported on how to care for a newborn baby after birth, particularly effective breastfeeding practices after delivery. Therefore, their judgement of quality of care and satisfaction was based on the level at which such expectations were met.

Findings from this study indicated that some women (53, 80.3%) actually received better care than they expected, while (60, 90%) stated that all their expectations were met. This could probably be the reason behind the report of high satisfaction obtained from this research. Also, the chi-square test of association between maternal expectation and satisfaction with perinatal care received indicated a statistically significant relationship. This result is similar to a previous study conducted by Galle, VanParys, Roelens, and Keygneart where the researchers discovered that women whose expectations were met expressed higher levels of satisfaction [24]. Fenny, Enemark, Asante, and Hansen, in a study on patient satisfaction in Ghana, also found that individual expectation influenced their perception of quality of care and their satisfaction with the care received [25]. Therefore, findings from this study further supports that maternal satisfaction is highly dependent on the extent to which maternal expectations are met by care providers.

### Relationship between Perceived Quality of Care and Women’s Satisfaction with Perinatal Care

This research revealed that there is a significant association between perception of quality of care and satisfaction with care (p < 0.05). Analysis of obtained data reveals that 98.5% of women rated the care received as high quality, and a corresponding 94% of the total respondents had a high level of satisfaction with care. This result is similar to the results obtained from a research conducted by Fenny, Enemark, Asante, and Hansen, where they reported that 98% of patients attending primary health care centres were satisfied with the quality of care they received [25].

### Relationship between Maternal Level of Education and Women’s Satisfaction with Perinatal Care

The results of this study indicate no significant association between maternal level of education and women’s satisfaction with care. This result is in contrast with previous study conducted by Ige and Nwachuckwu on areas of dissatisfaction with primary health care services in government-owned health facilities in Igboora community, a south-western, semi-urban community in Nigeria [4]. Ige and Nwachuckwu reported that having income above N20,000 monthly and having a tertiary level of education were factors found to be associated with dissatisfaction with quality of care [4]. In a similar study by Chemir, Alemseged, and Workneh in south-west Ethiopia, it was revealed that a majority of the respondents (76%) had no secondary education, and the respondents with lower level of education tend to have comparatively higher level of satisfaction than the respondents with some form of tertiary education [26].

### Relationship between Maternal Satisfaction with Perinatal Care and Likelihood for Subsequent Utilization of Primary Health Centres for Perinatal Care

This study found that there is an association between maternal satisfaction with quality of care and likelihood for subsequent utilization (Figure 1). The study revealed that a majority (75.8%) of the respondents have a high likelihood of subsequent utilization of the respective PHC for perinatal care, while 98.5% stated that they would recommend utilization of the PHC facility to others for quality perinatal care. This result is similar to previous studies locally and internationally, which asserted that maternal satisfaction with care is a major determinant for subsequent utilization of facility [6,27,28].

### Attitude of Health Care Providers towards Clients

The attitude of healthcare providers constitutes a major aspect of interpersonal relationship and has been identified as one of the major determinants of patients’ satisfaction. However, results from this study indicate that the respondents were satisfied with the attitude of staff in terms of respect, politeness, and kindness. Majority of the respondents (95.5%) agreed that the midwives and nurses had good attitude towards the patients. Sixty respondents (90.9%) agreed that the nurses were very respectful, and 93% of the respondents agreed that midwives always showed respect to their families. This respect could probably be a reason for the perception of care as high quality and the report of good satisfaction with care provided. This result is similar to the result of a previous study by Iloh et al. in south-eastern Nigeria, where the researchers reported that 81.5% of the respondents were satisfied with the attitude of the care provider and the patient-provider relationship [1]. A similar result was reported by Iliyasu et al. in a study on patient satisfaction in Kano (northern Nigeria), where the researcher reported that 87% of respondents were also satisfied with patient-provider interpersonal relationship [28].

### Areas of Dissatisfaction

Although the majority of the women perceived the quality of care as very high and satisfactory, there are certain aspects of care with which the women were not satisfied. For instance, about one-fifth of respondents (14 respondents, 21%) reported having been beaten at least once by a midwife at the primary health centre, and 16 respondents (24.2%) agreed to having been verbally abused at least once by a midwife during the course of receiving perinatal care in the PHC. It should be noted that on no basis should a nurse or midwife verbally or physically abuse a patient, irrespective of the situation or what the patient could have done wrong, because this is against the ethics of the nursing profession. Although it was very few, some of the respondents also reported having been shouted at by a midwife during the course of care.

Other factors that respondents identified as causes of dissatisfaction include dirty hospital environment, dirty toilet, inadequate water supply, distance of hospital location, increased cost of some services (e.g., hospital card, prescription of very expensive medications), having to pay for things hospital management is supposed to supply, time wasting, inadequate hospital facilities, inadequate beds, inadequate chairs in the waiting area, inadequate staffing (midwives are not enough), poor attitude, preferential treatment, and disrespect.

This result is similar to previous studies conducted on maternal satisfaction in Nigeria. Dzomeku iterated that poor attitude of caregivers such as ineffective communication (impolite, frowning, whispering, not explaining procedures), neglect (not attending to clients promptly, not involving client in the care), unfriendliness (shouting, ignoring), and financial factors (no explanation on hospital bills), amongst others, are major factors that make women uncomfortable [12]. Iliyasu et al., in a study on patients’ satisfaction in Kano, similarly reported that major causes of dissatisfaction as stated by respondents included time wasting and high cost of care [28].

### Implication of Findings to Nursing

Findings from this study further emphasises the importance of maternal satisfaction to utilization of health facility. It has been revealed that satisfaction is largely dependent on the extent to which expectations are met. It is therefore imperative to note that every woman approaching the primary health centre has some fundamental expectations about quality of care to be provided, and the extent to which these expectations are met determines the level of satisfaction and likelihood of subsequent utilization of health facility. Hence, midwives should pay attention to women’s needs and provide opportunities for them to state how they feel and be carried along with their care. All women expect to be treated with respect and be given adequate information to make informed decision as regards the care of pregnancy, the process of delivery, and care of the baby after delivery.

This study has revealed that aside from competence and skilfulness of the midwives, the attitude of care providers and interpersonal relationship between the midwives and the patient constitute the basis of maternal perception of quality of care and their level of satisfaction with care. Therefore in order to maintain the high level of maternal satisfaction and continually improve the quality of care at the primary health centres, midwives should make a conscious effort to provide patient-centred, individualized care. Midwives should treat the mothers with respect; communicate effectively; provide encouragement, support, praise; and give quality health education. Midwives should advocate for a conducive environment as well as the provision of necessary facilities for quality care.

## Conclusion

Patient satisfaction has been identified as a major index in the assessment of quality of healthcare globally. Mothers judge the quality of perinatal care received based on their satisfaction with the services provided, thus influencing their utilization of the available health facilities. There is currently a dearth of literature on users’ satisfaction of services rendered at a primary level of care, which is the first port of call to the health system aimed at serving the majority of the populace – thus the need to investigate maternal satisfaction with perinatal care received in selected primary health centres in Ibadan. The cross-sectional survey involved 66 women receiving postpartum care in Ibadan North-West Local Government.

Findings revealed that the majority of respondents perceived the perinatal care they received as of high quality and were satisfied with services and facilities used for their care. Identified causes of dissatisfaction included dirty hospital environment, inadequate water supply and hospital facilities, distance of hospital location, cost of materials, time wasting, inadequate staffing, poor attitude, and verbal and physical abuse. An association was found to exist between maternal satisfaction with quality of care and future intention for subsequent utilization.

Though the overall maternal satisfaction on the perinatal care provided was very good, few domains of dissatisfaction identified need to be addressed by healthcare professionals and policymakers to sustain and improve utilisation of orthodox health care services amongst mothers, thereby contributing to achieving the third Sustainable Development Goal.

Care should be timely with respect, devoid of any form of abuse, and targeted towards providing quality care as well as meeting the needs and expectations of the women, with caregivers displaying competence and adequate skills.

## Limitations of This Study

Social desirability bias could have affected the quality of data collected because some respondents had difficulty in expressing dissatisfaction in the presence of others, despite the fact that the researcher has explained that their responses would be totally confidential. Also, the five primary health centres are located quite far from each other, and hence the research consumed a lot of money in terms of transportation to the research sites.

## Recommendations

There is a need to institute measures for measuring patients’ satisfaction in all public primary health centres. This will provide a basis for assessment of quality of care from users’ perspective and provide roadmap for improving quality of care.

There is need for more in-depth research on the factors affecting adequate utilization of primary health centres amongst mothers in higher socio-economic class in Nigeria.

In conclusion, the role of government in improving quality of care in public primary health centres cannot be over-emphasized. Government should provide adequate facilities and conducive environment for quality health care delivery. Government should make proper provision for adequate staffing and funding for the health sector.
